# Efficacy and safety of Shu-gan-qing-re formula for generalized anxiety disorder: study protocol for a multi-center, prospective, double-blind, double-dummy, randomized controlled trial

**DOI:** 10.1186/s13063-020-4186-6

**Published:** 2020-03-14

**Authors:** Pei Chen, Hong Zhu, Yanzhe Ning, Dongqing Yin, Hongxiao Jia

**Affiliations:** The National Clinical Research Center for Mental Disorders & Beijing Key Laboratory of Mental Disorders, Beijing Anding Hospital, Capital Medical University & The Advanced Innovation Center for Human Brain Protection, Capital Medical University, No. 5, Ankang Hutong, Xicheng District, Beijing, 100088 China

**Keywords:** Generalized anxiety disorder (GAD), Shu-gan-qing-re (SGQR) formula, Traditional Chinese medicine (TCM)

## Abstract

**Background:**

Generalized anxiety disorder (GAD) is a persistent and common mental disorder that entails significant impairments in role functioning and quality of life. Currently available effective interventions include psychological therapies, self-help approaches, and pharmacological treatments, which do not quite meet clinical needs, and the ideal anxiolytic is still being sought. Shu-gan-qing-re (SGQR) formula, a Chinese patent medicine, has been well received by patients with GAD in Chinese clinical practice for years. The present prospective, double-blind, double-dummy, randomized controlled trial is designed to investigate the efficacy and safety of SGQR formula for GAD.

**Methods/design:**

A total of 200 eligible participants will be recruited from four hospitals in different parts of China. They will be randomly assigned to either the study group or the control group in a ratio of 1:1. Participants allocated to the study group will receive SGQR formula and buspirone placebo, while buspirone and SGQR placebo will be applied in the control group. Six scheduled visits will be conducted over the course of 8 weeks. Outcome measurements include Hamilton Anxiety Rating Scale (HAM-A), Hamilton Depression Rating Scale-17 (HAMD-17), Clinical Global Impression-Improvement Scale (CGI-I), Traditional Chinese Medicine Syndrome Scale for GAD, and pro-inflammatory cytokine tests: interleukin-1 beta (IL-1β), IL-6, and tumor necrosis factor-alpha. Adverse reactions will be evaluated by using the Treatment Emergent Symptom Scale (TESS). Safety outcomes and adverse events will also be recorded.

**Discussion:**

The study will provide scientific and objective assessments for the efficacy and safety of SGQR formula for patients with GAD, hopefully offering clinicians an alternative approach to GAD.

**Trial registration:**

Chinese Clinical Trial Registry, ID: ChiCTR-IPR-17013058. Registered on October 20, 2017.

## Background

Generalized anxiety disorder (GAD) is a persistent and common disorder characterized by feelings of threat, restlessness, irritability, sleep disturbance, tension, and symptoms such as palpitations, dry mouth, and sweating [1]. An epidemiological study in China suggested that the illness has a lifetime prevalence of 0.3% and a 12-month prevalence of 0.2% (the highest prevalence is in the 50- to 64-year-old group) but shows no gender difference [2]. The high prevalence of concurrent psychiatric disorders makes the diagnosis of GAD difficult, possibly contributing to the different epidemiological results of China, Europe, and the US [3, 4]. Comorbidity with at least one other mental disorder at some point troubles the majority of patients with GAD, most commonly with major depressive disorder [1, 5]. Besides, GAD is associated with considerable economic costs owing to work productivity loss and high medical resource use. The role impairments of pure GAD are similar in magnitude to those of major depressive disorder [5].

GAD has a relapsing course, and treatments rarely result in complete resolution of symptoms. In the short to medium term, effective intervention includes psychological therapies, such as cognitive behavioral therapy; self-help approaches based on cognitive behavioral therapy principles; and pharmacological treatments [1]. Until the mid-1980s, pharmacological treatment options consisted primarily of benzodiazepines, which later became an effective short-term therapy in consideration of their abuse potential. For patients requiring long-term treatment, buspirone and some newer antidepressants became the alternatives. Nevertheless, the mild efficacy combined with a slow onset of action dampened the satisfaction with buspirone, whereas antidepressants, mainly selective serotonin reuptake inhibitors (including paroxetine), brought both efficacy and lots of adverse effects [6]. The search for the ideal anxiolytic has never stopped.

Traditional Chinese medicine (TCM) is the most popular alternative and complementary medicine in China. It has a long history in the treatment of mental disorders and has favorable clinical effectiveness. According to the theory of TCM, anxiety results mainly from the stagnation of the liver qi, and inner heat emerges as a result. Shu-gan-qing-re (SGQR) formula is tailored from famous TCM prescriptions: Xiao-yao powder and Gan-mai-da-zao decoction. It is composed of Radix Paeoniae Alba, Radix Bupleuri, Angelica Sinensis, and seven other herbals and functions by smoothing the liver qi. SGQR formula can help to relieve anxiety and has been used in Chinese clinical practice for years.

The present multi-center, prospective, double-blind, double-dummy, randomized controlled trial is designed to investigate the efficacy and safety of SGQR formula, setting buspirone as the control treatment. We hope that, through this rigorously designed study, we can provide scientific and objective assessments for the efficacy and safety of SGQR formula for patients with GAD and possibly offer the world an alternative anxiolytic.

## Methods/design

### Study design

**Fig. 1:**
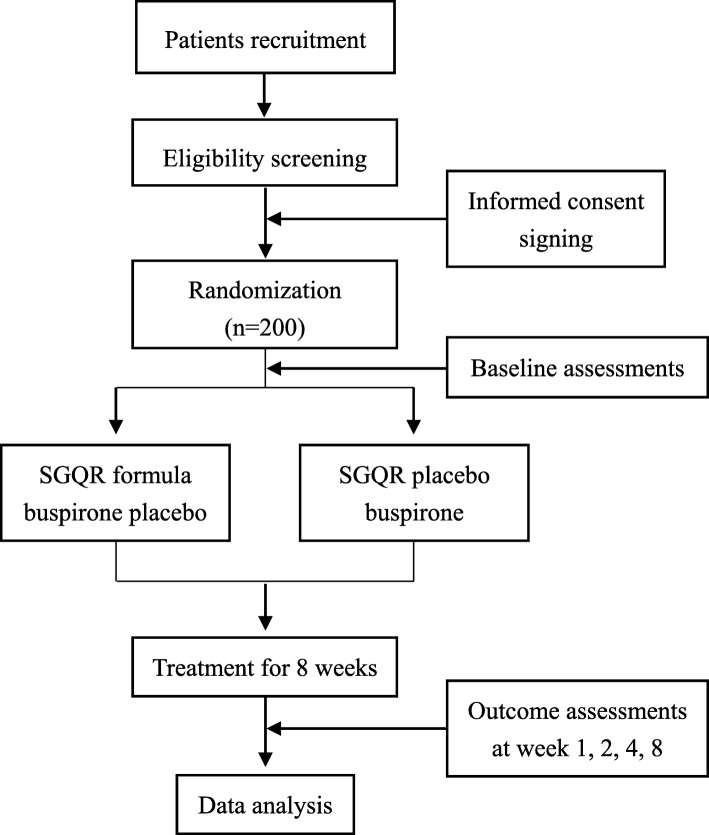
Study design

This is a multi-center, prospective, double-blind, double-dummy, randomized controlled trial. The flowchart of the study is presented in Fig. 1.

### Ethical issues

The clinical trial has been registered on the Chinese Clinical Trial Registry (ChiCTR-IPR-17013058). The study protocol (version 2.0 in August 2017) has been approved by the research ethics committee of Beijing Anding Hospital, Capital Medical University ((2017)63-201772FS-2). The research ethics committee will also be responsible for supervising all procedures of the study, including participant recruitment, randomization, conduction, and data storage. In case of any changes to the study protocol, we will submit a written application to the research ethics committee. They will decide whether it is acceptable to make the change.

### Participant recruitment

A total of 200 eligible participants will be recruited from four hospitals in different parts of China: Beijing Anding Hospital, Xiamen Xianyue Hospital, Tangshan 5th Hospital, and Zhumadian 2nd People’s Hospital. Both inpatients and outpatients will be screened for participation from November 2017.

### Informed consent

Potentially eligible patients will be invited to take part in our study. Prior to enrollment, researchers will fully introduce the detailed procedures of the study to the patients. All questions and concerns raised about the study will be addressed at length. Patients will also be informed of the probable benefits and potential risks and assured that participation is entirely voluntary. Patients who meet all of the inclusion criteria and none of the exclusion criteria will be enrolled after providing written informed consent. The personal information of all the participants will always be kept confidential.

### Criteria

#### Inclusion criteria

Participants meeting all of the following criteria will be enrolled: (1) inpatients or outpatients with GAD diagnosed in accordance with the International Classification of Disease-10; (2) those with a diagnosed TCM pattern of liver-qi stagnation transforming into fire, which usually manifests as irritability, constriction in the chest, hypochondriac distention, gastric discomfort with acid regurgitation, dry mouth or bitter taste in the mouth (or both), constipation, headache, conjunctival congestion, tinnitus, reddened tongue with yellow fur, and rapid pulse; (3) ages between 18 and 65 (no limitation on gender); (4) Hamilton Anxiety Rating Scale (HAM-A) scores between 14 and 39 (inclusive) at screening and baseline; and (5) written informed consent obtained either personally or by proxy.

#### Exclusion criteria

Participants meeting any one of the exclusive criteria will not be enrolled: (1) patients with serious suicidal tendencies; (2) patients with glaucoma; with severe or unstable internal medical conditions involving mainly the heart, liver, kidney, endocrine system, or the blood; or with diseases that may induce acute or chronic abnormal levels of pro-inflammatory cytokines (systemic lupus erythematosus, ulcerative colitis, etc.); (3) history of epilepsy (hyperpyretic convulsion in the childhood excepted); (4) alcohol or drug dependence in the previous year; (5) anxiety resulting from other mental or physical disorders; (6) pregnant or breastfeeding women or women who plan to conceive during the study period; (7) history of severe drug allergy or allergy to buspirone; (8) participation in other clinical drug trials in the previous 30 days; (9) intake of monoamine oxidase inhibitor or buspirone in the previous 4 weeks; (10) regular medicine intake cannot be guaranteed because of being uncared or other reasons; (11) a reduction of the HAM-A score of at least 25% at baseline compared with the screening time; and (12) a Hamilton Depression Rating Scale-17 (HAMD-17) score of more than 7 at screening.

### Randomization and allocation concealment

Eligible participants will be randomly assigned to either the study group or the control group in a ratio of 1:1. A computer-generated random allocation sequence will be produced by an independent statistician using SAS software (SAS Institute, Cary, NC, USA). Based on the allocation sequence, drug packages will be labeled serially. Participants will receive the corresponding drug package in the order of recruitment. The complete random sequence will be kept by the primary researcher in Beijing Anding Hospital. Emergency envelopes containing the random allocation individually will be prepared for each center, which can be unsealed only in the case of serious adverse events.

### Blinding

Pharmacists in charge of labeling the drug packages do not take part in the conduction of the trial. Participants and researchers responsible for screening, recruitment, dispensing medicine, and outcome assessment will all be blind to the group allocation. Statistical analysis will be conducted by independent statisticians who will know only the group codes but not the corresponding interventions.

### Interventions

Participants allocated to the study group will receive SGQR formula and buspirone placebo, while buspirone and SGQR placebo will be applied in the control group. The drug and its matching placebo will have a similar appearance, weight, and taste. SGQR formula and placebos will be provided by Tiansheng Taifeng Pharm Co. Ltd. (Lhasa, Tibet, China), and buspirone will be manufactured by Enhua Pharm Co. Ltd. (Xuzhou, Jiangsu, China).

SGQR formula is prepared in the form of condensed pills. The pills will be packed at a unit of 4 grams, whose ingredient is equivalent to Radix Paeoniae Alba 1.14 g, Radix Bupleuri 0.86 g, Angelica Sinensis 0.57 g, Radix Curcumae 0.57 g, Poria Cocos 0.69 g, Bulbus Lilii 0.69 g, Cortex Albiziae 0.69 g, Radix Glycyrrhizae 0.34 g, *Triticum aestivum* L 0.86 g, and Fructus Jujubae 0.57 g, 10 herbals in total.

There will be three dosages of drugs: the starting dosage (SGQR formula or placebo 4 g t.i.d., combined with buspirone or its placebo 5 mg b.i.d.), the low dosage (SGQR formula or placebo 4 g t.i.d., combined with buspirone or its placebo 5 mg t.i.d.), and the high dosage (SGQR formula or placebo 8 g t.i.d., combined with buspirone or its placebo 10 mg t.i.d.).

In the first week of intervention, participants will be prescribed the starting dosage for the first three days and the low dosage for the remaining four days. From the second week on, the dosage will be adjusted by the researchers. The recommended indicator for doubling to high dosage is a reduction of not more than 25% of the HAM-A score compared with the baseline after the first week’s intervention, keeping low dosage otherwise. Moreover, high dosage and low dosage can be alternated anytime during the intervention period in accordance with patient’s reaction to the previous treatment.

Drugs and placebos covering the whole course of treatment for each participant will be packed uniformly and individually. In each center, there will be a pharmacist responsible for dispensing the prescription. At every visit, participants will receive the drugs scheduled for the next intervention period, and the drugs left from the previous period will be returned. The dosage will be explained to the participants orally and in writing and also recorded in a medical booklet. Medication compliance will be calculated accordingly. The scheduled duration of treatment is 8 weeks unless a serious adverse event happens or the participant quits. Non-pharmacologic care is permitted but other psychiatric medication is prohibited. Administration and dosage of concomitant medication for physical conditions will be permitted and recorded in detail.

**Fig. 2:**
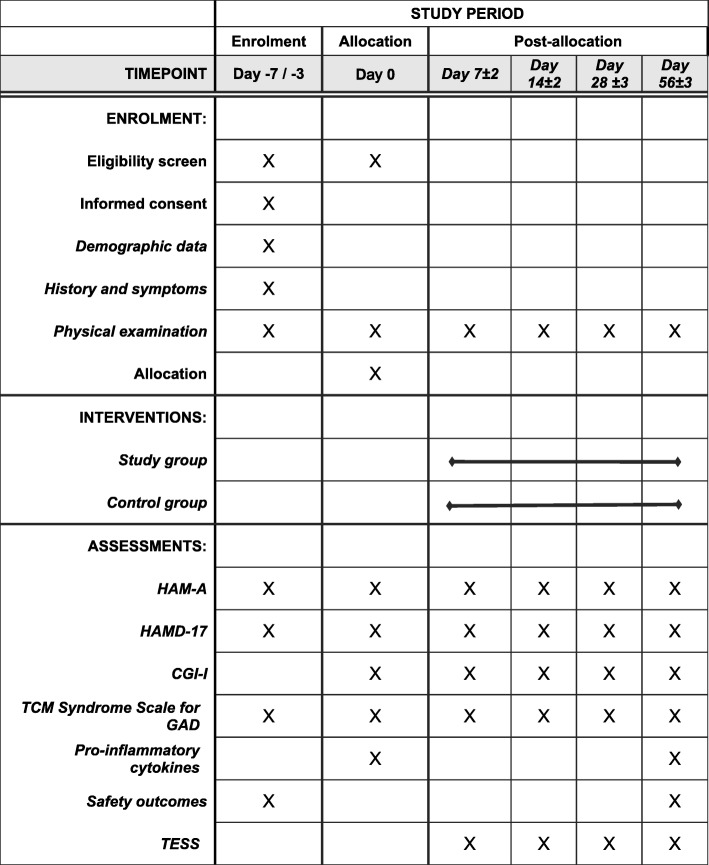
Study schedule

Any question raised by the participants will be answered to promote completion. The detailed study schedule is listed in Fig. 2.

### Outcome measurements

Outcome measurements are scheduled at screening, baseline, and the end of weeks 1, 2, 4, and 8.

#### Basic characteristic variables

Basic data of all the participants, including name, gender, date of birth, marital status, educational level, date of diagnosis, and medical history, will be collected. General physical examination results, including vital signs (body temperature, blood pressure, respiratory rate, and heart rate), will also be recorded. Concomitant medication will be inquired about and documented at every visit.

#### Primary outcome

HAM-A, which is one of the first and most widely used rating scales to measure the severity of anxiety symptoms, will be set as the primary outcome [7]. There are 14 items in the HAM-A, which incorporate groups of symptoms (such as fears, autonomic symptoms, and respiratory symptoms) rather than specific, single symptoms. Each item is rated from 0 to 4; the higher the score, the worse the situation. The good reliability and validity of HAM-A make it a standard interviewer-administered instrument [8].

#### Secondary outcomes

HAMD-17 is set as one of the secondary outcomes because comorbidity of depression and anxiety is rather common in health-care settings [9]. HAMD-17, a multi-dimensional rating scale that covers a range of clinical features associated with depression, is the standard efficacy outcome for depression severity evaluation, treatment response, and remission measurement [10, 11]. The higher the HAMD-17 score, the worse the situation.

Another secondary outcome focuses on the improvement in overall symptom severity and functioning, measured by using the Clinical Global Impression-Improvement Scale (CGI-I), which consists of a 7-point global rating (1 = very much improved, 4 = no change, and 7 = very much worse) [12]. This scale has also been widely used in clinical studies on GAD.

The TCM Syndrome Scale for GAD is a tailored scale for GAD and is based on the theory of TCM. It concentrates more on subjective discomfort in combination with objective clinical signs. Subjective discomfort includes worry without reason, hesitation, being easily frightened, and a disinclination to talk because of a deficiency of qi. These symptoms may not be fatal but always trouble the patient seriously. Objective clinical signs focus on the complexion, color of the lips and tongue, tongue coating, and the pulse. When the patient is evaluated with this scale, the differentiation and severity of the TCM syndrome can be concluded.

#### Therapeutic mechanism outcomes

Pro-inflammatory cytokines, including interleukin-1 beta (IL-1β), IL-6, and tumor necrosis factor-alpha, will be tested at baseline and the last visit. An altered cytokine balance (that is, a relatively increased pro-inflammatory response and decreased anti-inflammatory response) was found in GAD [13]. Previous clinical study showed that the anxiolytic effects of selective serotonin reuptake inhibitors may depend on their anti-inflammatory effects, proved by the reduced levels of pro-inflammatory cytokines after treatment [14]. Whether SGQR formula also has an effect on the immune balance needs to be investigated. All blood samples will be collected and centralized for tests.

#### Safety outcomes

Safety outcomes involve complete blood count, electrolyte panel, liver and kidney function tests, urinalysis, urine pregnancy test, and electrocardiograph. Safety outcomes will be assessed at screening time and the last visit.

### Adverse events

Adverse reactions will be checked by using the Treatment Emergent Symptom Scale (TESS) at every visit [15]. If any adverse events happen during the treatment period, all details, including the time of occurrence, clinical symptoms and signs, degree, duration, laboratory findings, treatment, outcomes, and causal relationship with the treatment, will be recorded in the case report forms (CRFs). Serious adverse events will be reported to the research ethics committee, which will decide whether any additional measures should be taken.

### Quality control

Before the conduction of the protocol, a range of training will be provided to all the researchers, ensuring a full understanding and mastery of the standard operating procedures. Any change to the protocol should be reported in writing to the primary researcher in Anding Hospital and can be applied only after permission. The quality of CRFs will be regularly supervised by independent investigators in Anding Hospital. Researchers will be notified in a timely fashion if any error exists.

### Sample size calculation

As there is no large randomized controlled trial of SGQR formula for GAD, we referred to the experience of experts in this field. A response rate of 91% for SGQR was assumed. As to buspirone, the response rate was about 75%, according to the literature [16]. In the setting of α = 0.05 and β = 0.2, the sample size was calculated to be 83 in each group. Given a 20% dropout rate, 17 more participants will be required in each group. In total, 200 participants will be enrolled.

### Data management and analysis

CRFs will be entered by two independent researchers and supervised by a third one to ensure accuracy. Data analysis will be performed by statisticians who are blind to the randomization by using SPSS (Statistical Product and Service Solutions) Statistics for Windows, version 20.0 (IBM Corp., Armonk, NY, USA). Continuous variables will be presented as mean and standard deviation, and categorical variables will be presented as frequency or percentage. Demographic data and outcome measurements at baseline will be listed to ensure relatively even baseline levels. A *t* test (if normally distributed) or non-parametric test (if not normally distributed) will be used for continuous variables when conducting baseline comparisons. The repeated measures ANOVA (analysis of variance) will be used when analyzing the primary and secondary outcomes. Chi-squared or non-parametric test will be applied for categorical variables. The analysis of efficacy will be performed in accordance with the intention-to-treat principle. Missing values will be imputed by the last observation carried forward method. Subgroup analysis will be conducted if necessary. A *P* value of less than 0.05 will be considered statistically significant. A mixed-effects model for repeated measures will be performed as a sensitivity analysis of the primary efficacy outcome.

Mid-term date analysis will be conducted after half of the recruitment is finished, and the principal investigator will decide whether any measure should be taken in light of the results. All of the data will be deposited in the platform developed by our team. Anding Hospital will be responsible for the data, and other researchers can have access to the data only after application and permission.

## Discussion

Just like many other Chinese patent medicines, SGQR formula has been widely used in China for years, but strong clinical evidence supporting its use is still lacking, limiting its global popularization. Buspirone, a 5-hydroxytryptamine 1A receptor agonist belonging to azapirones, has been approved for the treatment of GAD in adults and has been shown to be effective [17, 18]. A Cochrane review of randomized controlled trials of azapirones in adults with GAD indicated that this class of medications is relatively well tolerated and effective [18, 19].

In this study, we chose the commonly used buspirone as the control and set a number of scientific and objective assessments. We hope that the results of this clinical trial, if successfully conducted, will provide a promising approach to treating GAD.

## Trial status

The protocol version was 2.0 (August 2017). Participant recruitment began in January 2018, is ongoing at the time of this writing, and will be finished by about June 2020.

## Data Availability

The authors declare that all relevant data will be included in the article or supplementary files. Additional data are available from the corresponding author on reasonable request.

## Abbreviations

b.i.d.: Twice a day
CGI-I: Clinical Global Impression-Global Improvement Scale
CRF: Case report form
GAD: Generalized anxiety disorder
HAM-A: Hamilton Anxiety Rating Scale
HAMD: Hamilton Depression Rating Scale
IL: Interleukin
SGQR: Shu-gan-qing-re
TCM: Traditional Chinese medicine
TESS: Treatment Emergent Symptom Scale
t.i.d.: Three times a day
